# Examining Ferrihydrite Nanomaterials: From Structure and Biosynthesis to Biomedical and Agricultural Applications

**DOI:** 10.3390/nano16201302

**Published:** 2026-10-14

**Authors:** Alexei M. Grachev, Sergey V. Stolyar, Alla I. Perfileva, Yuri L. Gurevich, Natalia G. Menzyanova, Margarita I. Teremova, Tatyana R. Krikunova, Valentina P. Ladygina, Ana Pantelica, Claudia G. Chilom, Maria Balasoiu

**Affiliations:** 1 Krasnoyarsk Science Center, Federal Research Center KSC SB RAS, 660036 Krasnoyarsk, Russia; stol@iph.krasn.ru (S.V.S.); mennage@mail.ru (N.G.M.); mterem@mail.ru (M.I.T.); tkrikunova95@mail.ru (T.R.K.); ladvp@mail.ru (V.P.L.); 2 Kirensky Institute of Physics, Federal Research Center KSC SB RAS, 660036 Krasnoyarsk, Russia; 3 Siberian Federal University, 660041 Krasnoyarsk, Russia; 4 Siberian Institute of Plant Physiology and Biochemistry, Siberian Branch of Russian Academy of Sciences, 664033 Irkutsk, Russia; alla.light@mail.ru; 5 Horia Hulubei National Institute for R&D in Physics and Nuclear Engineering, 077125 Magurele, Romaniamasha.balasoiu@gmail.com (M.B.); 6 Faculty of Physics, University of Bucharest, 077125 Magurele, Romania; claudia-gabriela.chilom@unibuc.ro; 7 Joint Institute for Nuclear Research, 141980 Dubna, Russia; 8 Research-Development Center for Intelligent Materials and Biomedical Applications, West University of Timișoara, 300223 Timișoara, Romania

**Keywords:** ferrihydrite, nanoparticles, structure, green synthesis, biomedical applications, agriculture, SAXS, SANS, IBA techniques (PIXE, PIGE, RBS), elemental composition, *Klebsiella oxytoca*, nanozyme

## Abstract

This multidisciplinary work is an attempt to synthesize advances in ferrihydrite (Fh, Fe_10_O_14_(OH)_2_·nH_2_O) research and applications ranging from physics to nanoagriculture. Fh nanoforms obtained via chemical or “green” synthesis methods using various bacterial cultures display unique catalytic and enzyme-like properties, surface reactivity, and magnetic properties. SAXS and SANS, supported by model-dependent fitting (FITTER) and model-independent ATSAS tools (Guinier approximation, *p*(*r*) function, and ab initio reconstruction), together with Porod-law and fractal analysis, provide a coherent multi-scale picture. Biogenic nanoparticles produced by *Klebsiella oxytoca* are built from 1–2 nm crystallites embedded in an organic matrix and exhibit a cultivation-dependent, hierarchical architecture: e.g., Fe12 yields a two-level hierarchical structure, whereas Fe34 produces a sponge-like surface. SANS further reveals that ferrihydrite nanoparticles in complex with lactate dehydrogenase form a core–shell bionanocomposite. The practical applications lie primarily in the biomedical field and plant cultivation, with significant implications for medicine and food production. A more thorough understanding of the underlying mechanisms, as well as studies to determine precise dosages and application methods, is critical. Investigating the biological activity of Fh NPs under both physiological and pathological conditions will facilitate the development of biologically informed engineering strategies for new Fh-based agents aimed at treating serious human diseases, improving plant mineral nutrition, protecting plants from phytopathogens, and remediating soil. Future directions include engineering nano-agrochemicals by functionalizing nanoforms tailored to specific crop types and soils with distinct physicochemical properties.

## 1. Introduction

Rapidly advancing applied nanoscience in the area of synthetic metal-containing nanomaterials is fundamentally based on the fact that, in contrast to conventional macroscopic particles with constant properties, nanoparticles (NPs) exhibit variable properties related to the large relative fraction of surface atoms. With demonstrated extraordinary physicochemical characteristics, the diversity of engineered NPs is now immense, and they are finding expanding technological applications across a wide range of fields, from food production to medicine [1,2]. Generally, nanomaterials can be (1) natural; (2) synthesized via physical/chemical pathways; or (3) biosynthesized, produced via “green synthesis”. While the current scientific literature on nanomaterials is dominated by laboratory-engineered substances that have no natural analogs, some naturally occurring nanomaterials remain overlooked.

Nanomineral ferrihydrite (Fh) is an iron (Fe(III)) oxyhydroxide with a formula conditionally designated here as Fe_10_O_14_(OH)_2_·nH_2_O. It may be considered from the point of view of geochemistry as “one of the most important iron-containing minerals on Earth” [3]. However, in contrast to common iron oxides, Fh has garnered quite limited attention, primarily due to the challenges associated with its characterization. Fh can be referred to as a pronounced example of a *pur sang* nanomaterial [4]. While all Fe(III) forms are characterized by very low solubility, Fh is somewhat more soluble, with solubility increasing in the following order: hematite < goethite < lepidocrocite < Fh [5]. The discovery of Fh was very recent, relatively speaking—this hard-to-identify compound received its name only in 1971 [6]. Among the recent key developments, the activity of Fh as a nanozyme has been independently demonstrated in several studies, primarily with activity similar to that of superoxide dismutase and catalase. Ma et al. [7] showed that the iron cores of biogenic ferritins are effective in scavenging superoxide radicals within organisms. These authors emphasize that, given the high stability and ease of modifying ferritin structures, the identification of superoxide dismutase-like activity in natural ferritin nanozymes offers a novel strategy for addressing superoxide-related diseases such as rheumatoid arthritis and osteoarthritis and mitigating damage from radiotherapy and chemotherapy. Zhang et al. [8] argued for catalase-like activity instead. The efficacy of ferrihydrites as catalase-like nanozymes for enhancing radiotherapy sensitivity showed the critical influence of abundant iron-associated hydroxyl groups on the surface. Unlike other iron oxyhydroxides, Fh not only lacks a precisely defined composition but is also characterized by variable physicochemical properties. Fh particle sizes approach the lowest nanoscale limit, with nearly half of all Fe atoms in NPs being the surface atoms, as emphasized by [4]. Fh is characterized by a very large specific surface area that may exceed 600 m^2^/g [9] and is characterized by abundant adsorption sites and reactive OH sites on the surface [10]. Being widespread in the natural environment (soils, cold-water springs, thermal springs, sediments, etc.), Fh is a quintessential nanosized iron mineral. Whether chemically synthesized or of natural origin, it can only exist in the form of NPs, typically 2–6 nm in size. Other prominent examples of ubiquitous nanomaterials ever-present in the environment include clay minerals and some sulfides [11]. Leaving more details to the subsequent sections, indeed, “Fh is a puzzling nanosized mineral with a 50-year-old history of debates over its structure, composition, and formation mechanisms” [10]. The most authoritative review remains that of J. Jambor and J. Dutrizac [12], containing a thorough examination of the physical and chemical properties of Fh based on studies from the 1990s and earlier.

The structural organization of ferrihydrite, its high surface reactivity with active OH radicals, and its magnetic response enable a remarkably extensive range of documented functions. Correspondingly, the biological activity of ferrihydrite has been demonstrated in numerous studies, outpacing the understanding of the mechanisms of its action. Future research is necessary to explore the interrelationships among structural characteristics, material properties, and functional performance. Predictable performance characteristics of various forms of Fh (synthesized chemically or by various bacteria, doped, etc.) are needed for both biomedical and agricultural research. The present work summarizes recent essential research on Fh, including its green synthesis; provides an overview of structural studies; and highlights several venues for applications, including biomedicine and agriculture.

## 2. Green Synthesis of NPs

Green synthesis of nanoparticles is currently positioned as an eco-friendly alternative to traditional physicochemical methods of NP synthesis [13,14]. Numerous studies indicate the feasibility of both intracellular and extracellular NP synthesis using cultures of bacteria, fungi, and microalgae, as well as extracts from higher plants [13,14,15,16,17]. However, there remains a challenge in achieving a comprehensive understanding of the underlying biochemical processes governing nucleation kinetics, surface stabilization mechanisms, crystallinity, morphology, and surface chemical properties [14,18,19,20,21]. Species-specific metabolic characteristics determine differences in the size and morphology of synthesized NPs when identical precursors are used [18]. Variations in physicochemical characteristics have been observed in NPs produced in bacterial cultures under different cultivation conditions. Life cycle characteristics, aging processes, and the general variability of biological systems also influence the reproducibility of the resulting NP physicochemical properties [18,19].

Examples of iron-containing NPs obtained via green synthesis include FeO NPs synthesized using aqueous extracts of amaranth leaves [21], elemental Fe NPs obtained on a plant extract with *Urtica dioica* L. or tea polyphenols (*Camellia sinensis* L.) [13,22], and Fe_2_O_3_ and Fe_3_O_4_ NPs synthesized using an extract of date palm leaves (*Phoenix dactylifera* L.) [1], among many others (see, e.g., [14]). It is well established that the properties of chemically synthesized NPs of various metals, for example, their adsorption properties, may differ significantly from those of biosynthesized NPs [23].

The reproducibility of green synthesis results is not merely a technical challenge complicating the development of quantitative models and scale-up procedures. Resolving this challenge will elevate green synthesis technologies to a qualitatively new level. There is no doubt that various forms of biological variability drive the biochemical diversity of NP synthesis processes, even within a single species. Consequently, the biological variability inherent in green synthesis—while technologically inconvenient—may serve as the foundation for the controlled bioengineering of diverse nanoforms from a single precursor.

Targeted regulation of the producer organism’s physiological and biochemical processes will enable the production of NPs with specific physicochemical characteristics tailored to address particular practical challenges. The directions include (1) increasing crop yields (via nanofertilizers, nanopesticides, and nanoadaptogens); (2) targeted drug delivery in medicine; (3) regulating redox homeostasis at the cellular level; and (4) modifying genomes, intracellular signaling systems, and gene expression patterns. Targeted NP bioengineering will facilitate the accurate assessment of dose-dependent effects and the potential toxicity of specific nanoforms within the biosystems.

In the case of ferrihydrite, the prospects for bioengineering various nanoforms depend not only on using different bacteria and varying cultivation conditions. There are prospects of genetically modifying the producer organism and employing co-cultivation systems that may yield entirely new metabolite profiles.

## 3. Ferrihydrite

### 3.1. Early Studies

The molecular architecture of Fh presents a challenge for X-ray crystallographic identification due to its fundamental characteristic: the absence of long-range crystal order. Contrary to some initial views, the phase corresponding to Fh is not amorphous and is not characterized by a simple composition of Fe(OH)_3_: “Fe(III) hydroxide”, discussed in some early publications, originating from the natural environment or of synthetic origin, was none other than Fh. It could be designated by authors in general terms, such as “colloidal Fe(III) oxide/hydroxide”, “hydrated Fe(III) oxide”, or sometimes simply as “Fe(III) hydroxide”, and be described as amorphous or paracrystalline [12,24].

Formally, the discovery of Fh may be dated to 1967, when K. Towe and W. Bradley characterized it in detail using physical methods [24]. Subsequently, in 1971, with reference to this foundational work, the name “ferrihydrite” for this newly thoroughly studied weakly crystalline phase with low X-ray diffraction properties was introduced [6]. It was noted that chemically synthesized Fh clearly corresponded to one specific mineral widespread in the natural environment. As noted in a subsequent paper [25], Fh is a geologically ephemeral material that can be considered “protohematite” from a geochemical viewpoint. Essentially, Fh, which lacks long-range order, is a transitional form between an amorphous compound and hematite, characterized by familiar crystalline properties with long-range order. Adsorption of various ions has a stabilizing effect on Fh, slowing down its natural geochemical transformation into other mineral forms [12].

### 3.2. Structure

The exact structure of Fh remained highly controversial despite the fact that very sophisticated instrumental methods were used, in addition to the standard suite generally used in mineral characterization [26,27]. After decades of research, various chemical formulas have been proposed for Fh based on an idealized three-dimensional structure, including some fairly simple ones, such as 5Fe_2_O_3_·9H_2_O (e.g., [28]), and others more complex, such as Fe_5_O_8_H·nH_2_O [29], Fe_10_O_14_(OH)_2_ or Fe_10_O_14_(OH)_2_·H_2_O [26,30,31] and Fe_10_O_14_(OH)_2_·nH_2_O [32]. We will not dwell in more detail on the various variants of the chemical formula for Fh, noting only that the previous debates and variability in the formula are basically inherent to Fh and are not due to methodological problems or the role of its originating source. Following studies by F. Michel et al. [26] and others, it is generally accepted today that Fh can be considered an isostructural analog of an aluminum mineral: Al_10_O_14_(OH)_2_ (akdalaite).

**Figure 1:**
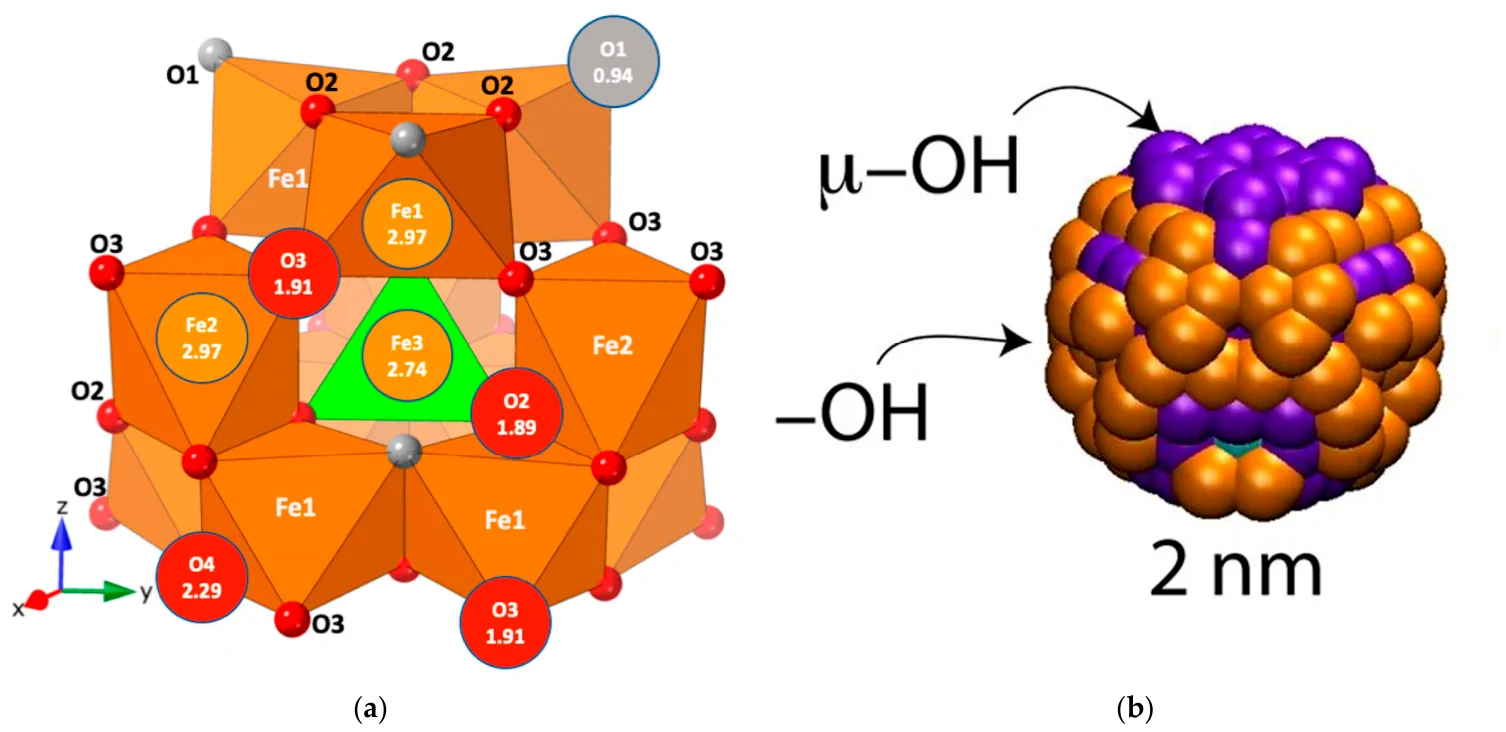
Structure of Fh. (**a**) General structure of Fh (from [35]; see also [36]). The revised polyhedral structure model features the Keggin motif with the calculated bond valences. The octahedral Fe is depicted in orange; the tetrahedral Fe is in the center (shown in green). Oxygen atoms are shown in red, and the hydroxyl oxygen atoms (O1) are represented in gray. The ideal bond valence sums for oxygen and hydroxyl are 2 and 1, respectively, while for iron sites, it is 3 (see [35,36]). (**b**) Chemically active surface of a model Fh nanoparticle with abundant OH-sites, which can be coordinated differently: singly (FeOH, –OH), shown in brown; doubly (Fe_2_OH, μ-OH), shown in purple; and triply (Fe_3_OH, μ_3_-OH), shown in turquoise (only one site is seen) (from [10]; see also [37]).

The coordination of Fe in minerals is critical to its crystal structure. In early studies, there were suggestions that the internal structure of Fe in Fh is octahedral, while on the surface, it is tetrahedral [12]. Parallels between the structure of Fh and that of wustite (FeO) and hematite (*α*-Fe_2_O_3_) were noted early on, with its crystallization state increasing during the natural transformation of ferrihydrite into hematite (see [33] and references therein). In modern understanding, Fh can be described to a good approximation by a hexagonal model with tetrahedral and octahedral Fe [26,34]. The structural unit cell in a simplified form can be represented as shown in Figure 1a. The central atom in the crystal lattice is tetrahedral Fe; the surrounding oxygen atoms are not equivalent [26,35,36]. The surface of Fh is extremely reactive due to abundant OH-groups [10] (Figure 1b).

According to the generally accepted single-phase model, the mineral phase of Fh must include both tetrahedrally and octahedrally coordinated Fe [9]. The proportion is one tetrahedral Fe in Fh for 12 octahedral atoms, with several studies confirming the presence of tetrahedral Fe [3,38]. A good description is provided by an ensemble of several individual Fh NPs, embedded in an amorphous yet structured, to a certain degree, non-random Fh matrix. While there is no long-range order, short- and intermediate-range order is present. Fh nanocrystals coexist with the amorphous matrix, as they have a similar chemical composition, and there are three types of interactions that must be considered for accurate description: nanocrystal–nanocrystal, matrix–matrix, and nanocrystal–matrix; without such an approach, unrealistic values for bond lengths are obtained [3].

The variable composition of Fh is inherent to the nature of this nanomaterial: this is due to its weak crystal ordering (the absence of long-range order), its configuration/conformation of the three-dimensional structure realized during formation, and the amount of bound H_2_O (variable hydration). Over a long time prior to 1967 [24], these factors have hampered the identification of Fh. Furthermore, significant changes in the properties of Fh can be observed in the presence of impurities. For example, Si content can exceed 30% in natural Fh [27], with some properties changing dramatically as a result compared to pure Fh nanomaterial. An additional factor is that the individual grains formed are always nanosized, regardless of the synthesis method or natural source. Natural Fh in the environment, in reality, may be a physical mixture of individual nanoscale phases with different chemical compositions. Examples of pure Fh in nature are very rare [39].

### 3.3. Types of Fh

#### 3.3.1. Chemically Synthesized Fh

The purely chemical synthesis of Fh has long been known and does not present any difficulties [40]; traditionally, it was carried out by hydrolysis [12]. The “solvent-deficient method” of synthesis proposed in the work of Smith et al. [30], based on Fe(NO_3_)_3_·9H_2_O and NH_4_HCO_3_, is quite simple and does not require strict control of reaction conditions. Particles in the narrow size range of 2–6 nm are obtained using that method.

#### 3.3.2. Biosynthesized Fh

Following an expedition in the late 1990s, by around 2000, a technique for isolating and culturing certain bacteria from lake sediments in the Krasnoyarsk region (closed-basin Lake Borovoe–Plakhino) with the potential to produce Fh was established. The subsequently developed biosynthesis method allowed for the reproducible production of stable Fh nanomaterial with Fe citrate as one possible substrate. The method initially involved the use of a magnetic separator to select a bacterial species, which was subsequently identified as heterotrophic *Klebsiella oxytoca*, which can exist both in the presence and absence of oxygen. Biosynthesized Fh is inherently not identical to the chemically synthesized form due to its biogenic origin and multiple reactions taking place over time within the complex bioorganic medium [41,42,43,44]. Importantly, a characteristic polysaccharide shell is formed in the case of Fh biosynthesized on *K. oxytoca* (e.g., [45,46]). The first publications on Fh NPs obtained from *K. oxytoca* were pioneering for their time and corresponded to “green synthesis”. They began to appear only after the accumulation and interpretation of Fh results using Mössbauer spectroscopy and other physical methods starting in 2005 [41,47,48,49]. It should be noted that other *K. oxytoca* strains were isolated by two other research groups in Italy and in Iran, albeit with objectives other than the precise identification of the biosynthesized Fe compound ([45,50,51,52,53], etc.).

Subsequently, alternative methods of producing Fh using aerobic heterotrophic bacteria *Delftia tsuruhatensis*, *Pseudomonas moorei*, *Bacillus aryabhattai*, and *Acinetobacter calcoaceticus* were also developed [43]. The resulting Fh is characterized as a “nanocomposite with a high organic carbon content” [54]. For Fh synthesized in the laboratory using bacteria, the term “bacterial Fh” or “biogenic Fh” has traditionally been used [54,55,56]; hereafter, we refer to it as “Bio-Fh”. We are aware of only a few studies in which Fh biosynthesis on bacteria different from *K. oxytoca* and *D. tsuruhatensis* has been reported by other research groups: Chukhrov et al. [25] employed *Gallionella ferruginea* bacteria; Ahmed et al. [57] employed *Comamonas testosteroni*; marine phototrophic Fe(II) oxidizer *Rhodovulum iodosum* was used by Byrne and Kappler [58]; and a culture of *Ralstonia pickettii* isolated from a mineral spring produced Fh was reported by Kianpour et al. [59].

Additionally, Fh can be produced from ferritin of different origins [7,34]: Michel et al. [34] showed that Fh of animal origin, isolated from ferritin, exhibits a significantly reduced tendency to aggregate. This is contrary to chemically synthesized Fh, which forms aggregates in aqueous solution, leading to the loss of important properties, potentially significantly limiting access to the particle surface and reducing reactivity.

#### 3.3.3. Two-Line and Six-Line Fh

In addition to the general structural variability inherent in Fh, two distinct modifications are distinguished. The number of X-ray diffraction peaks, two or six, identifies two Fh end-members, with intermediate forms between them occurring in natural environments [60]. The six-line type represents a more crystalline form, and the two-line type has a minimal degree of crystallinity [61]. The latter is characteristic of natural conditions and bacterial synthesis. The two-line type is characterized by a smaller average NP size and by higher magnetization values. Sassi et al. [62] pointed out that the two-line and six-line Fh phases may actually comprise an assemblage of distinct particle types not readily distinguishable by X-ray diffraction or X-ray scattering.

### 3.4. General Significance of Fh

The general significance of Fh from the point of view of life sciences and environmental sciences is very briefly outlined here. It is well established that Fh plays a key role in the storage and metabolism of Fe, supporting vital processes in virtually all living organisms. It is not present in the organism by itself but rather as part of the protein ferritin, enclosed in a complex shell, as was demonstrated back in the 1980s [63]. An early evolutionary development, the ferritin shell is composed of amino acid chains organized in a spherical capsule [64]. Iron is stored as Fh in the cavity in ferritin of around 8 nm [65].

In the natural environment, Fh occurs in the context of ongoing or geologically recent geochemical processes. These processes can occur in near-surface soils, mineral springs, and sediments, where Fe generally occurs in two forms: extremely poorly soluble Fe(III) compounds and highly soluble Fe(II) compounds. Fh is typically found only in moist and cool conditions, while other mineral forms, primarily goethite and hematite, dominate under other conditions. Examples of various natural objects containing Fh were first presented in the work of Chukhrov et al. [25]. The authors noted that the environment in all studied objects was characterized by a pH close to neutral, a narrow temperature range (5–7 °C), and the presence of specific bacterial species. As discovered recently, Fh is the principal iron oxide constituent of Martian dust, contradicting earlier hypotheses that anhydrous minerals were dominant [29].

Fh is relatively mobile; it plays a significant role in the global Fe biogeochemical cycle. It is the least crystallized form of Fe(III), which is the most soluble and very reactive. In the ocean, diatoms are able to accumulate Fe, which indirectly affects the magnitude of primary carbon production on a global scale [66]. On land, Fh serves as a repository of bioavailable ferric iron for bacterial microorganisms. Fh is an important component not only in the biogeochemical cycle of Fe itself but also in the cycles of heavy metals that are actively bound to it upon contact (e.g., [67]). In summary, Fh still remains “a key yet misunderstood iron oxyhydroxide nanomineral in natural sciences” [10], with great significance across many disciplines.

### 3.5. Scope of Physical Studies on Bio-Fh

An essential property of Bio-Fh synthesized by *K. oxytoca* is the presence of a specific polysaccharide shell encapsulating Fh [51]. This feature shares similarity with the protein ferritin, which encapsulates Fe in the form of Fh in its central cavity. It is possible to use water-soluble proteins or natural polysaccharide arabinogalactan as a stabilizing matrix [68]. The authors of the first studies on Bio-Fh particularly noted the nearly identical magnetic properties of Bio-Fh and ferritin. Numerous studies have been devoted to the structure and properties of Bio-Fh NPs obtained specifically from *K. oxytoca* [28,46,55,69,70,71,72,73,74,75,76] employing a wide range of advanced methods. NPs of Fh biosynthesized on *K. oxytoca* consist of a dense antiferromagnetic core and a loose shell [46]. Anghel et al. [77] evaluated structure and particle morphology, the stability of water dispersions, and chemical composition and performed morphological and structural analyses. Balaev et al. [78] studied magnetic properties and their dependence on temperature and superparamagnetic behavior. Knyazev et al. [79], in addition to magnetic properties, studied NP size distribution in detail. Magnetic properties were further studied using an originally designed instrument for pulsed magnetic field measurements by Krasikov et al. [80]. It has also been demonstrated that Bio-Fh NPs synthesized using *C. testosteroni* bacteria exhibit peroxidase-like activity and catalyze the reactions of chromogenic peroxidase agents [57].

## 4. Advanced Structural Studies

The present section systematizes the results of structural studies (scanning electron microscopy (SEM), high-resolution transmission electron microscopy (HRTEM), and primarily small-angle X-ray and neutron scattering) of two different types of ferrihydrite nanoparticles produced either chemically, via alkaline hydrolysis of iron salts, or biogenically, through the metabolism of microorganisms, such as the bacterium *K. oxytoca*. Biogenic ferrihydrite nanoparticles, in turn, were of two distinct types depending on cultivation conditions: growth duration, illumination, potassium citrate/ferric citrate ratio, etc. (Fe12 and Fe34 samples).

### 4.1. Cultivation Conditions, Sample Nomenclature, and Elemental Composition

#### 4.1.1. Cultivation Conditions and Sample Nomenclature

Both biogenic specimens discussed throughout this review were obtained from the same bacterial biomass and differ solely in the duration of cultivation. The strain *K. oxytoca* isolated from the sapropel of Lake Borovoe–Plakhino was grown under microaerophilic conditions on Lovley medium of the following composition (g/L): NaHCO_3_ 2.5; CaCl_2_·H_2_O 0.1; KCl 0.1; NH_4_Cl 1.5; NaH_2_PO_4_·H_2_O 0.6. The ferric citrate concentration varied between 0.2 and 5 g/L, the yeast extract concentration was 0.05 g/L, and the benzoic acid concentration varied between 0.2 and 0.5 g/L; the potassium citrate/ferric citrate ratio and the illumination regime also varied [70,71]. Sample Fe12 was separated from the biomass after 8 days of incubation, and sample Fe34 after 21 days [68,70,71]. These designations are therefore operational rather than compositional labels. The visual difference between the two powders—the reddish-brown color of Fe12, typical of an Fe(III) oxyhydroxide, versus the grayish-green color of Fe34—makes independent verification of their chemical similarity a necessity rather than a formality.

The notations Fe12 and Fe34 derive from Mössbauer spectroscopy, which resolves non-equivalent Fe^3+^ positions:

- Fe(1) and Fe(2)—Slightly and moderately distorted octahedra of single and double layers (QS ≈ 0.5–1.1 mm/s);
- Fe(3) and Fe(4)—Strongly distorted positions corresponding to structural defects and nanoparticles near the protein shell (QS ≈ 1.5–1.8 mm/s).

When positions with similar parameters are combined, two phases are distinguished: Fe(12), the more ordered one, whose magnetic susceptibility is three times higher, and Fe(34), the more defective one, which serves as a nucleation center for hematite during heat treatment. Hereinafter, sample designations are provided as numerals (Fe12 and Fe34), while Mössbauer components and combined phases are given in parentheses (Fe(1)–Fe(4), Fe(12), and Fe(34)).

#### 4.1.2. Elemental Composition of Fe12 and Fe34 from Ion Beam Analysis

To verify that the two specimens do not differ substantially in chemical composition, both samples were examined by three complementary ion beam analysis (IBA) techniques carried out simultaneously at the 3 MV Tandetron accelerator of the Horia Hulubei National Institute for R&D in Physics and Nuclear Engineering using a 3 MeV proton beam: particle-induced X-ray emission (PIXE), particle-induced gamma-ray emission (PIGE), and Rutherford backscattering spectrometry (RBS) [71].

PIXE identified 14 elements in the examined samples (Table 1). The major constituents are Fe, P, Cl, K, and Ca, ranging from from tens of percent to the level of one percent; the minor elements Al, Si, S, Rb, Hg, and Pb are found at tenths of a percent, while Mn, Cr, and Zn were detected at the ppm level. PIGE independently confirmed P, Cl, and Fe and additionally quantified Na and Mg, which are not accessible by PIXE under the present experimental conditions (Table 2). RBS determined the elements C, N, O, P, Cl, K, Ca, and Fe; in addition, H was assessed with the help of the RUMP simulation code in fitting the experimental spectrum (Table 3). The common elements determined by PIXE, PIGE and RBS were found to be in agreement [70,71].

The PIGE values for Fe agree with the corresponding PIXE values within the 5–7% analytical uncertainties, providing a cross-validation of the two methods. The PIGE values for Cl and P are systematically ~15–35% lower than the PIXE values, with analytical uncertainties of ~13–35% and 5–6%, respectively; this discrepancy is within the stated analytical uncertainties of the relative standardization procedure for the thick-target geometry used.

#### 4.1.3. Comparison of the Two Specimens and Implications

The most relevant conclusion of the present review is that the two specimens are chemically very similar in their iron and oxygen content while differing markedly in their organic and halide fractions. In particular:

*Fe:O ratio, by RBS.* Expressed as a mass ratio, Fe:O is 24.6: 35.9 ≈ 0.69 for Fe12 and 25.9: 36.7 ≈ 0.71 for Fe34 (Table 3). In atomic terms, Fe:O is 5.6: 28.2 ≈ 0.20 and 6.1: 30.4 ≈ 0.20, respectively. The two specimens therefore have an essentially identical Fe:O stoichiometry, close to that expected for a ferrihydrite-like Fe(III) oxyhydroxide core. This is the strongest available evidence against any significant partial reduction of Fe^3+^ into Fe^2+^ in the Fe34 specimen: a reduction in even a fraction of the iron would be expected to alter the oxygen balance appreciably.

*Carbon content.* The RBS-derived carbon mass fraction is ≈1.5 times higher in Fe34 (11.9 wt.%) than in Fe12 (8.12 wt.%), consistent with a thicker or more carbon-rich organic (polysaccharide) matrix in the longer-cultivated specimen (Table 3) [70,71].

Regarding the Fe:O:C ratio specifically, the RBS data (Table 3) give the full set of major constituents needed for this comparison: Fe 24.6/25.9, O 35.9/36.7, and C 8.12/11.9 wt.% for Fe12/Fe34.

*Hydrogen and nitrogen.* Fe12 contains ≈1.2 times more hydrogen and ≈2.4 times more nitrogen than Fe34, indicating a greater proportion of hydrated organic material and/or residual proteinaceous biomass in the shorter-cultivated specimen (Table 3) [70,71].

*Halogens and alkaline elements, by PIXE-PIGE.* Chlorine is ≈10 times more abundant in Fe12, and Na ≈3.4, Mg ≈1.7, K ≈1.6, and Ca ≈1.2 times, while Rb is about three times lower (Table 1 and Table 2) [70,71].

*Elemental enrichment for Fe34, by PIXE.* Fe (1.21×), P (1.23×), Si (1.27×), S (> 8×), and the trace metals Rb (3.0×), Pb (2.5×), Hg (2.1×), and Cr (> 2.7×) (Table 1 and Table 2) [70,71].

The enrichment of Fe34 in a group of metals (Fe, Rb, Pb, Hg, Cr), as well as in Si and sulfur, is consistent with the longer cultivation period, i.e., with a longer accumulation of both iron and accompanying trace elements from the culture medium, and with a more advanced stage of biomineralization. Conversely, the strong depletion of Fe34 in chlorine indicates that the two specimens were harvested at different stages of the culture medium evolution.

Taken together, the PIXE, PIGE, and RBS data demonstrate that Fe12 and Fe34 are chemically closely related materials with an identical Fe:O stoichiometry but distinctly different organic and halide fractions. The chemical difference between the two specimens is therefore quantitative rather than qualitative, and the striking difference in color between the two powders is most plausibly attributed to differences in particle size, aggregation state, surface hydration, and organic coating rather than to a change in the oxidation state of iron.

#### 4.1.4. Limitations and Required Further Verification

It must be emphasized that none of the three IBA techniques used in [71] are sensitive to the oxidation state of iron: PIXE, PIGE, and RBS yield elemental, not speciation, information. Consequently, the hypothesis of a partial Fe^3+^ → Fe^2+^ reduction in the Fe34 specimen, which could in principle be promoted by saccharides acting as reducing agents in the culture medium, cannot be definitively excluded on the basis of the IBA data alone. Direct speciation methods are required to settle this question; in particular:

^57^Fe Mössbauer spectroscopy, which is already the technique that discriminates most reliably between the Fe12 and Fe34 specimens [46,55] and can quantify any Fe^2+^ contribution through the isomer shift and quadrupole splitting parameters;

Fe K-edge XANES/EXAFS, which provides direct access to the average oxidation state and local coordination;

X-ray photoelectron spectroscopy (XPS) of the Fe 2p region, which is surface-sensitive and therefore particularly suited to detecting a reduced surface layer.

Since the observed Fe:O ratios of the two specimens are indistinguishable within experimental error, and since Mössbauer spectroscopy performed on these same specimens has previously been interpreted in terms of a single Fe(III) ferrihydrite phase with different particle sizes and degrees of crystallinity [46,55], the available evidence currently favors chemical similarity rather than a change in iron oxidation state. Nevertheless, a dedicated Mössbauer and XANES study of the Fe12/Fe34 pair, ideally together with the IBA data reported here, would provide a conclusive and self-consistent answer, and such measurements are proposed as the next step in this line of work.

### 4.2. On Small-Angle Scattering (SAS)

Small-angle scattering (SAS) is a powerful family of techniques for structural analysis of non-crystalline systems at length scales ranging from a few to hundreds of nanometers. It encompasses both small-angle X-ray scattering (SAXS) and small-angle neutron scattering (SANS). The fundamental scattering geometry and data analysis formalism are common to both techniques; the essential difference lies in the physical origin of contrast. In SAXS, contrast arises from the difference in electron density between the scattering particle and the medium. In SANS, contrast is determined by the difference in neutron scattering length density, which depends on the nuclear composition (and, for magnetic scattering, on the magnetization density). That makes SAXS and SANS highly complementary: SAXS is sensitive to electron-rich inorganic cores, while SANS, through isotopic substitution (e.g., H_2_O/D_2_O exchange), can selectively highlight organic components or probe magnetic structures. For a dilute monodisperse system of non-interacting particles, the scattering intensity I(Q) as a function of the scattering vector magnitude Q=(4π/λ)sin(θ/2) (where λ is the wavelength and 2θ is the scattering angle) is expressed as [81]:

(1) I(Q)=n⋅P(Q)

where n is the particle number density; P(Q) is the orientationally averaged particle form factor, $P(Q)={\left|F(Q)\right|}^{2}$; and F(Q) is the form factor amplitude. In the presence of interparticle correlations, the structure factor S(Q) is introduced:

(2) I(Q)=n⋅P(Q)⋅S(Q)

SAXS measurements were performed on a Bruker Nanostar instrument at the Institute of Synthetic Polymer Materials, RAS, Moscow. Depending on the study, the accessible Q-range was approximately 0.007–0.26 Å^−1^ [68,69,77]. SANS measurements were carried out on the YuMO time-of-flight small-angle spectrometer at the IBR-2 pulsed reactor (Joint Institute for Nuclear Research, Dubna) [82], covering a similar Q-range and providing complementary structural information on both dry powders and liquid dispersions [83,84].

Two complementary approaches to data analysis were employed: (1) model-dependent fitting using the FITTER software package [85,86,87] by fitting analytical form factors (a cylinder and elliptical cylinder) to the experimental data [68,69], and (2) model-independent analysis using the ATSAS package (PRIMUS, GNOM, and GASBOR) [88,89,90], including Guinier approximation, pair-distance distribution function *p*(*r*), and ab initio shape reconstruction [77].

#### 4.2.1. Guinier Approximation

In the low-Q region, the scattering intensity follows the Guinier law [91]:

(3) $$I(Q)=I(0)\exp(-Q^{2}R_{G}^{2}/3)$$

where $R_{G}$ is the radius of gyration, characterizing the distribution of scattering density around the particle’s center of mass. This estimate is shape-independent.

#### 4.2.2. Pair-Distance Distribution Function

The *p*(*r*) function describes the distribution of all pairwise distances between points within the scattering particle. It is computed by indirect Fourier transformation of the scattering curve using the GNOM program [92]. The radius of gyration is then obtained from *p*(*r*) as:

(4) $$R_{G}^{2}=\frac{\int_{0}^{D_{\max}}r^{2}p\left(r\right)dr}{2\int_{0}^{D_{\max}}p\left(r\right)dr}$$

where $D_{\max}$ is the maximum distance inside the particle. This method accounts for the entire experimental dataset, yielding more reliable *R_G_* values in the presence of minor aggregation [93].

#### 4.2.3. Ab Initio Shape Reconstruction

The GASBOR program [90] performs ab initio reconstruction of the molecular shape using a chain-like ensemble of dummy residues, producing a three-dimensional envelope.

#### 4.2.4. Porod Law and Fractal Scattering

At a large *Q* (relative to the particle size), the scattering from a system with sharp, smooth interfaces follows the Porod law [94,95]: *I*(*Q*) ∝ *Q*^−4^, corresponding to a scattering exponent α = 4. This *Q*^−4^ decay is the asymptotic signature of three-dimensional objects with well-defined interfaces and a specific surface area *S*/*V*.

Deviations from the *Q*^−4^ Porod behavior reveal interfacial or mass-fractal characteristics. For mass fractals, the scattering exponent satisfies 1 < α < 3, and the mass fractal dimension is *D_m_* = α. For surface fractals, 3 < α < 4, and the surface fractal dimension is *D_s_* = 6−α. A value of *D_s_* = 2 corresponds to a perfectly smooth surface (yielding the Porod limit), while *D_s_* → 3 indicates an extremely rough or convoluted surface.

These fractal descriptions are particularly relevant for biogenic ferrihydrite, where SAXS data from sample Fe34 [68] has revealed a power-law exponent of α = 3.18 ± 0.01, giving *D_s_* = 2.82 ± 0.01, a value characteristic of a highly convoluted, space-filling surface, consistent with the sponge-like morphology observed by SEM.

#### 4.2.5. Core–Shell and Multi-Level Models

When particles possess internal structure—such as a ferrihydrite core surrounded by an organic corona or a doped surface layer—the form factor must account for multiple scattering length density levels. For a spherical core–shell particle, the scattering amplitude is:

(5) $$F(Q)=\frac{1}{V_{tot}}\left[\left(\rho_{c}-\rho_{s}\right)V_{c}\Phi_{c}(Q)+\left(\rho_{sh}-\rho_{s}\right)V_{sh}\Phi_{sh}(Q)\right]$$

where $\rho_{c}$, $\rho_{sh}$, and $\rho_{s}$ are the scattering length densities of the core, shell, and solvent; $V_{c}$, $V_{sh}$, and $V_{tot}$ are the corresponding volumes of the core, shell, and total particle; $\Phi_{c}(Q)$ and $\Phi_{sh}(Q)$ are the normalized scattering amplitudes (form-factor functions) of the core and shell, respectively [81]. Analogous expressions exist for ellipsoidal and cylindrical geometries, which have been employed throughout ferrihydrite studies to model Fh NP aggregates, both pristine and doped with Co or Cu, and in complex with lactate dehydrogenase (LDH) [83,84], an enzyme found in almost all living organisms, whose main function is to catalyze the interconversion of lactate to pyruvate, based on the conversion of NAD into NADH.

### 4.3. Structural Characterization of Biogenic Ferrihydrite Nanoparticles

#### 4.3.1. SAXS Analysis of Biogenic Samples in Powder [68]

SAXS data for Fe12 and Fe34 were analyzed using the FITTER program [85]. Sample Fe12 exhibits two structural levels, as shown in Table 4.

**Figure 2:**
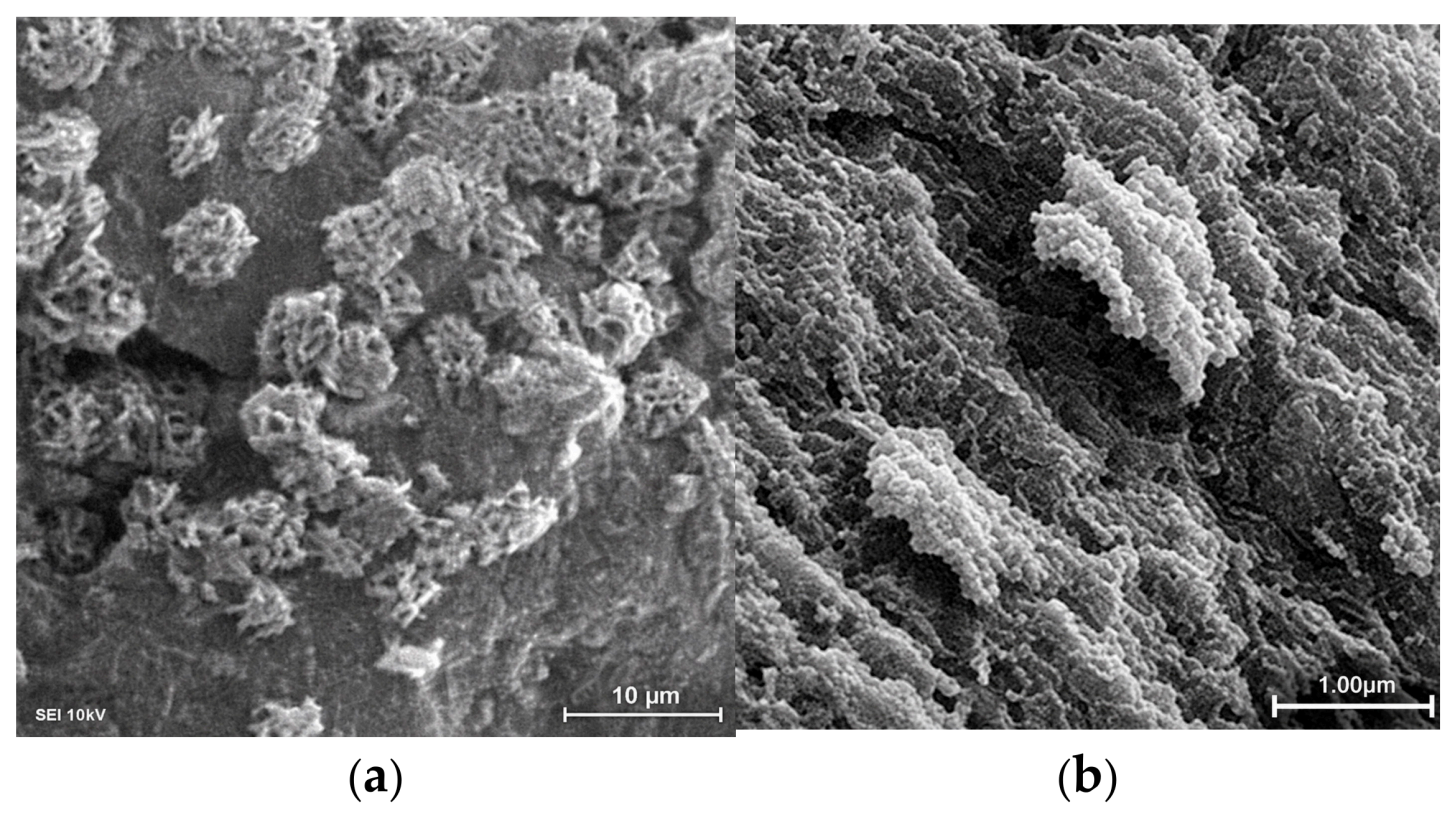
SEM images of Fe12 (**a**) and Fe34 (**b**) samples at magnifications of ×40,000 and ×30,000, respectively.

These results are consistent with SEM images (Figure 2) showing granular subunits that aggregate into larger elongated structures.

**Figure 3:**
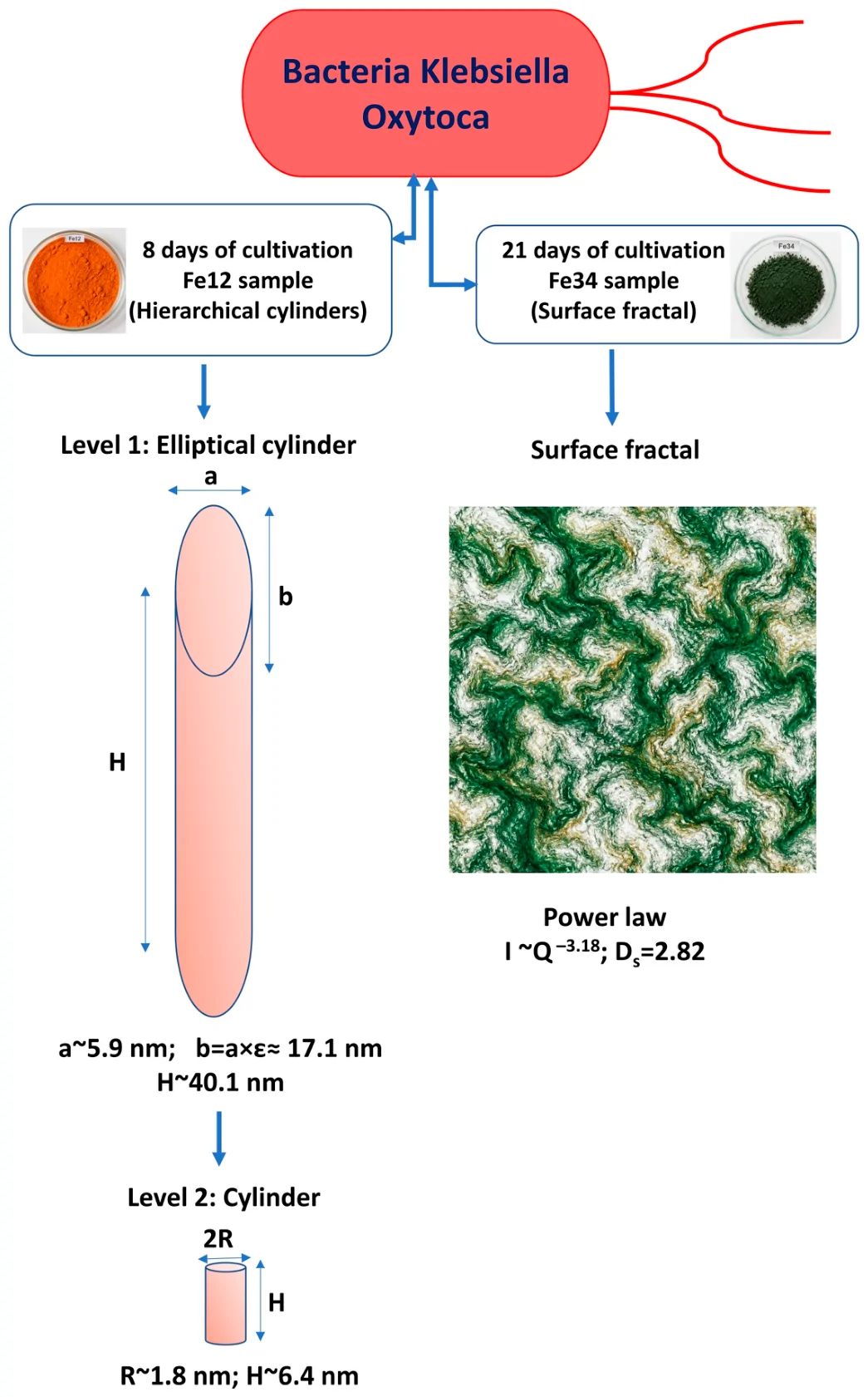
Model representation of the powder structures of Fe12 and Fe34 ferrihydrite samples synthesized by *K. Oxytoca* and obtained using the SAXS method. On the left is a two-level hierarchical cylinder model (8 days of cultivation); on the right is a surface fractal model (21 days of cultivation).

Sample Fe34 displays a markedly different scattering behavior: a power law $I(Q)\propto Q^{-\alpha }$ with exponent α = 3.18 ± 0.01, corresponding to a surface fractal dimension *D_s_* = 2.82 ± 0.01. This value is characteristic of highly branched surface fractals, in excellent agreement with the sponge-like, friable texture observed by SEM. Figure 3 shows a model image of the powder structures of Fe12 and Fe34 ferrihydrite samples synthesized by *K. oxytoca* and obtained using the small-angle X-ray scattering (SAXS) method.

#### 4.3.2. SAXS Analysis of Aqueous Dispersion of Fe12 Nanoparticles [77,96]

**Figure 4:**
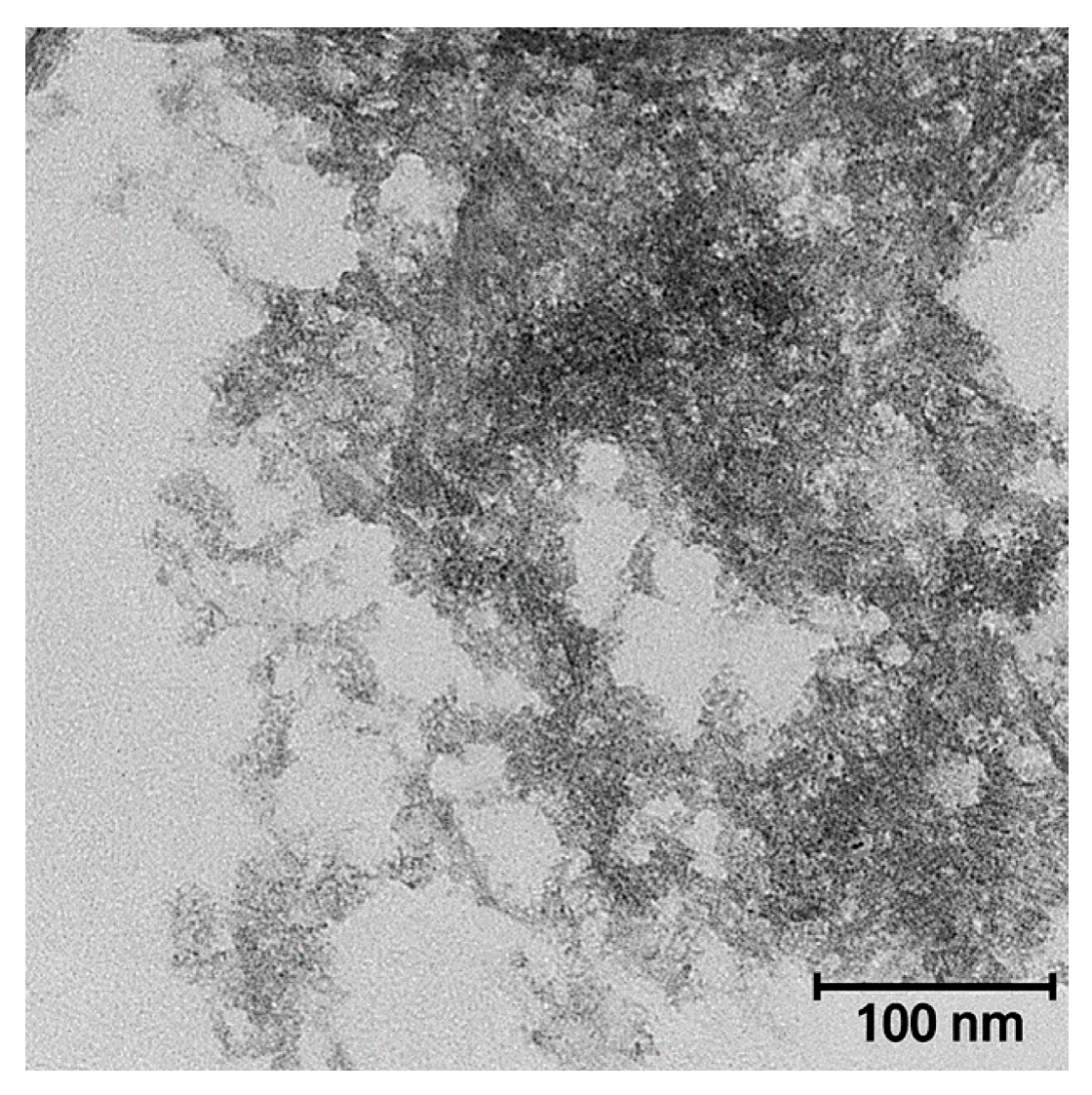
TEM image of Fe12 nanoparticle aqueous dispersion.

In aqueous dispersion (Figure 4), a fit with a cylinder form factor yielded a radius of *R* = 4.9 ± 0.02 nm and height *H* = 2.1 ± 0.04 nm, reflecting changes in particle organization upon hydration.

The radius increased by a factor of ~2.7 (from ~1.8 to ~4.9 nm), and the height decreased by more than a factor of 3 (from ~6.4 to ~2.1 nm).

Such a change in geometry may be attributed to the formation of a hydration shell, swelling of the organic matrix, and/or partial reorientation or fragmentation of aggregates upon dispersion [96].

The characteristic size of 1–2 nm determined by HRTEM is in good agreement with the cylinder height determined from SAXS. Similar particle size estimates (1.5÷1.8 nm) were independently obtained by the magnetogranulometry method from magnetization curve analysis [97].

For dilute, ultrasonicated ferrihydrite dispersions, a model-independent approach was applied at multiple concentrations.

To eliminate concentration-dependent effects and residual aggregation, a linear extrapolation to zero concentration was performed on scattering curves obtained at different ferrihydrite concentrations.

Guinier analysis yielded a radius of gyration *R_G_* = 6.4 ± 0.1 nm independently of any assumption about particle shape. A more refined estimate was obtained from the pair-distance distribution function *p*(*r*) calculated using the GNOM program, *R_G_* = 6.7 ± 0.2 nm. This value, derived from the full experimental dataset, is considered more reliable as it is less sensitive to minor aggregation effects than the Guinier estimate. The two values are in close agreement, confirming internal consistency [77].

**Figure 5:**
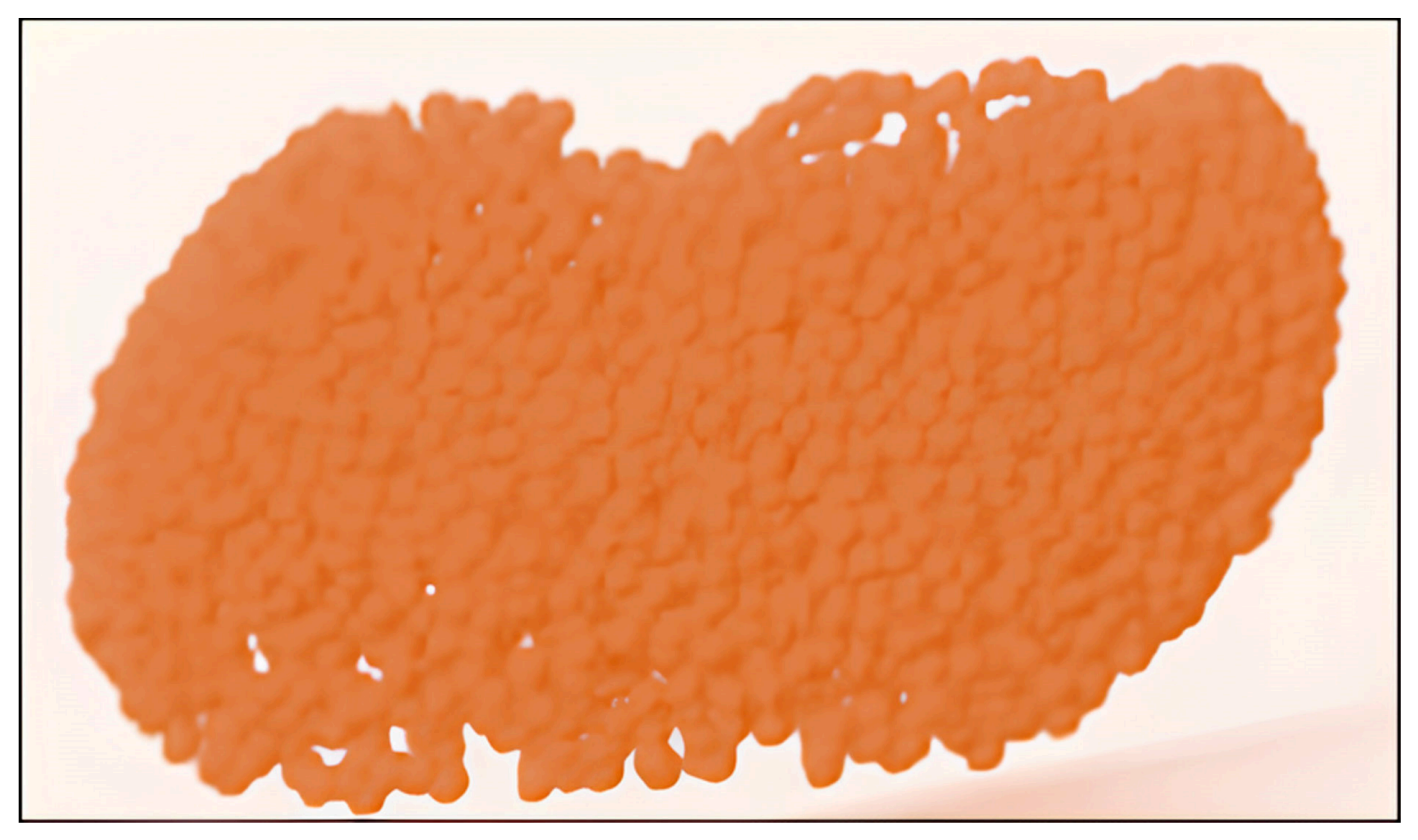
Ab initio reconstruction of ferrihydrite Fe12 nanoparticle in aqueous dispersion.

Ab initio shape reconstruction using the GASBOR program produced a 3D envelope with an elongated shape (see Figure 5) [77] that closely resembles the flat cylindrical model reported in the earlier concentrated-dispersion study [96]. While the reconstruction from 1D SAXS data is not unique—as is the case for any ab initio method—the agreement between the model-independent GASBOR shape and the earlier model-dependent cylindrical fit provides strong cross-validation.

**Figure 6:**
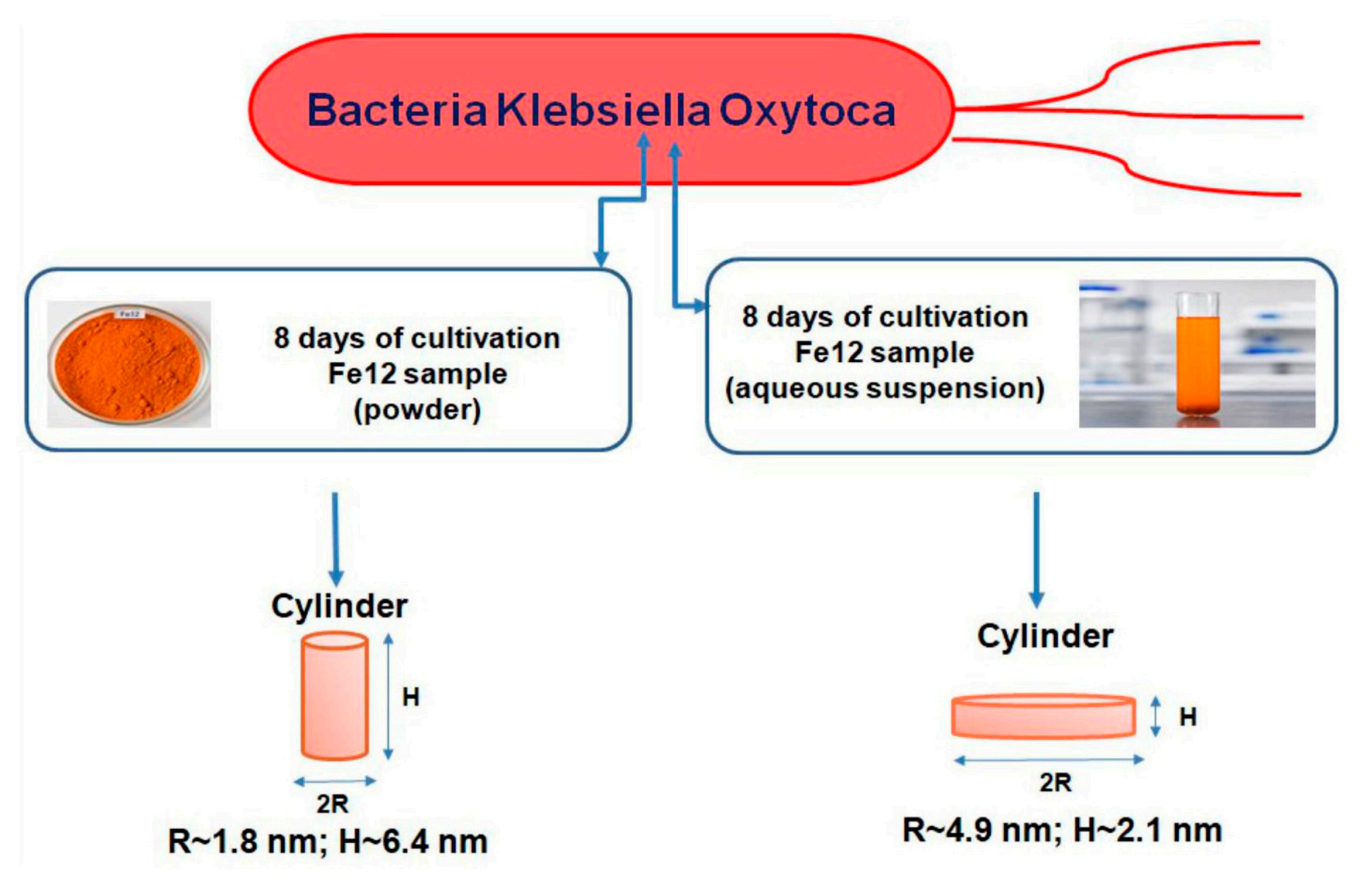
Comparison of the minimal structural unit of biogenic Fh NPs Fe12 in powder form and in aqueous solution, obtained by SAXS and model-dependent fitting with the FITTER software package.

Figure 6 shows a comparison of the minimal structural unit of biogenic Fh NPs Fe12 in powder form and in aqueous solution, obtained using SAXS and modeling with the FITTER software package.

### 4.4. Structural Characterization of Chemically Synthesized Ferrihydrite Nanoparticles

#### 4.4.1. SANS Analysis of Pure and Doped Ferrihydrite [84]

SANS measurements of dry powders of undoped, Co-doped, and Cu-doped Fh NPs prepared through a chemical precipitation method under precisely controlled ambient conditions as described in [72,73,74] were analyzed over the range Q = 0.007–0.05 Å^−1^. The experimental curves were fitted with geometric form-factor models [81]. The obtained structural parameters are shown in Table 5.

The minimal cluster dimensions (radii in the range of ~4.5–7 nm) are consistent with AFM particle heights, confirming that the primary particles aggregate into larger 3D clusters. The core–shell structure observed for doped particles suggests that doping may alter the aggregation mode, forming a distinct surface layer.

#### 4.4.2. SANS of Fh-NPs in Complex with Lactate Dehydrogenase

**Figure 7:**
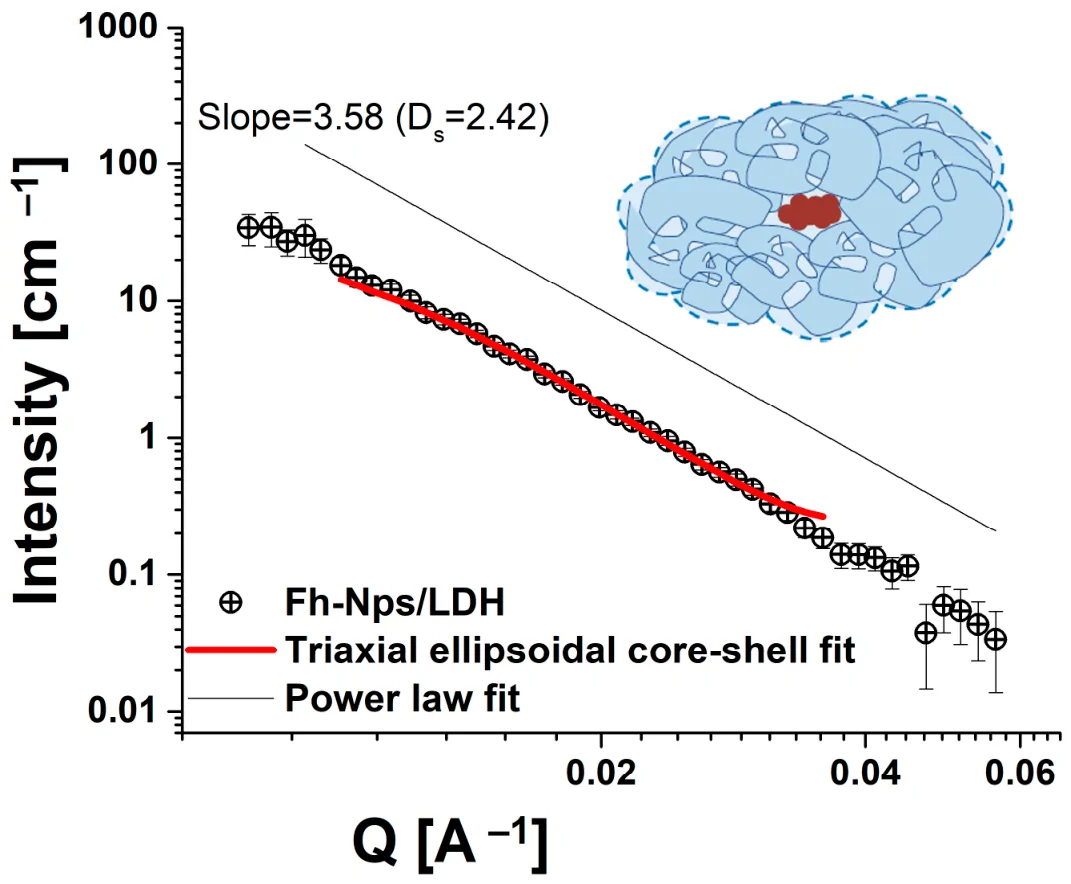
Experimental small-angle neutron scattering curves of the Fh NP/LDH system in suspension (black hollow circles), triaxial ellipsoidal core–shell model fitting curve (red line), standard power law fit (black line). Insert—schematic representation of the obtained model (core—Fh NPs; shell—LDH). Fitting was accomplished with FITTER.

SANS was further employed to characterize the structural organization of Fh NPs bound to the enzyme lactate dehydrogenase (LDH) [83]. The scattering curve of the Fh NPs/LDH complex in solution, after subtraction of the buffer contribution, was analyzed using three complementary approaches:

(i) The Guinier approximation, which works in the region of very small values of *Q* (*QR_G_* < 1.3), led to the determination of a structure with a radius of gyration R_G_~6 nm.
(ii) A linear standard power law plot over the mid-Q range yielded a surface fractal dimension *D_s_* = 2.42, indicating rough interfaces characteristic of protein–nanoparticle assemblies (see Figure 7)
(iii) The scattered objects were approximated as simple geometric bodies, and the data were fitted to an appropriately chosen multiparametric theoretical function (see Figure 7).

Fitting with a triaxial ellipsoidal core–shell model using the FITTER program gave the following structural parameters depicted in Table 6.

In this model, the core represents a cluster of Fh NPs, and the shell corresponds to the LDH protein corona. The overall dimensions of the Fh NPs/LDH complex remain in the submicron range (<500 nm).

Because the shell—a highly hydrated, swollen protein corona—has very low contrast with respect to the solvent, its contribution to scattering in the low-Q region is negligible. In this regime, the Guinier analysis effectively probes only the dense ferrihydrite core, yielding a radius of gyration *R_G_* ≈ 6.2 nm—a value characteristic of the inorganic nanoparticle itself. In contrast, the full-profile core–shell ellipsoid fit captures the contribution of the low-contrast organic corona, which extends to roughly 330 nm around a compact nanoparticle cluster. The two estimates thus probe different structural levels and are complementary rather than contradictory: the Guinier radius reflects the dense core, while the core–shell model resolves the extended, weakly scattering organic shell. This picture—a compact inorganic core embedded in a loosely packed, low-contrast protein corona—is consistent with the formation of a bionanocomposite whose overall dimensions may be further reduced by sonication or formulation optimization for biomedical applications.

### 4.5. Implications of Structural Features for Function

The structural distinctions of ferrihydrite nanoparticles revealed by small-angle scattering methods have direct functional consequences, outlined below.

Magnetic properties. The smaller primary crystallite size of biogenic Fh NPs (~1–2 nm) and their fractal organization influence the superantiferromagnetic response and the magnitude of the uncompensated magnetic moment, both of which are critical for magnetic manipulation in biomedical contexts.

Protein binding. SANS analysis of the Fh NPs/LDH complex demonstrates that chemically synthesized Fh NPs form a well-defined protein corona around ellipsoidal NP clusters. The fractal nature of the NP surface (*D_s_* = 2.42) may facilitate multivalent protein interactions. The triaxial ellipsoidal core–shell model rationalizes the entire set of experimental findings reported in [83]: the static quenching mechanism, the moderate binding affinity with an entropically driven hydrogen-bonding component, the FRET distance, the FTIR-detected secondary structure perturbations, and the thermal stabilization. Taken together, the irregular (fractal) core surface and the proteinaceous shell create a functional nanocomposite whose binding characteristics are well suited for biomedical applications.

Membrane interactions. The aggregation state and surface roughness characterized by SANS correlate with the ability of Fh NPs to modulate lipid membrane fluidity. The SANS structural parameters reveal a clear structure–function relationship [84]:

(i) Undoped Fh NPs form anisotropic triaxial ellipsoidal clusters that interact superficially with lipid head groups, causing mild rigidification without penetrating the bilayer.
(ii) Doping with Cu or Co creates a core–shell architecture—a ferrihydrite core surrounded by a thin (1.5–1.8 nm) dopant-enriched shell. This shell fundamentally alters the NP-membrane interaction mode, enabling penetration into the hydrophobic core.
(iii) Cu-Fh NPs (thicker shell, 1.80 nm) produce the strongest and most persistent membrane fluidization, consistent with Cu^2+^-mediated interactions at the bilayer interior.
(iv) Co-Fh NPs (thinner shell, 1.50 nm) show a more transient and cholesterol-dependent core penetration, with effects that diminish over time.
(v) The sub-10 nm size of all NPs (by TEM/AFM) permits passive insertion without endocytic machinery, while the SANS-detected larger cluster dimensions (tens of nanometers) reflect reversible agglomeration that sonication can disrupt—an important practical consideration for biomedical use.

Biocompatibility and toxicity. The presence of an organic corona in biogenic Fh NPs may enhance biocompatibility relative to bare chemically synthesized particles. Conversely, dopant metals (Co and Cu) in chemically synthesized particles introduce additional FTIR signatures and alter surface chemistry, with potential implications for cytotoxicity.

## 5. Biological Activity of Fh and Its Applications

### 5.1. Fh in Biomedical Applications of Fh

**Figure 8:**
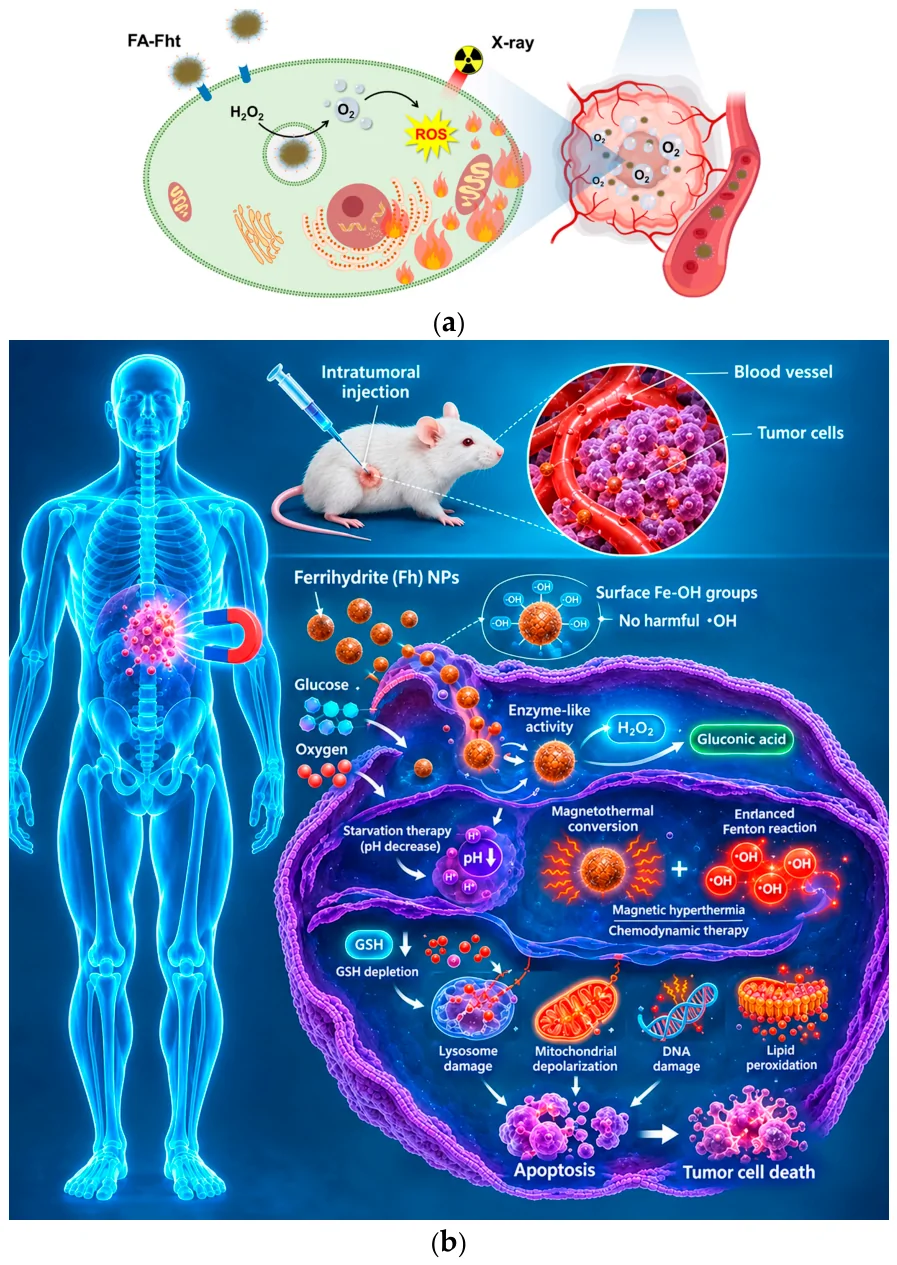
Targeting tumor cells. (**a**) The effect of Fh in reducing tumor hypoxia and enhancing radiotherapy (adopted from [8]). Fh significantly enhances the efficacy of radiotherapy in treating hypoxic tumors by expediting oxygen production at the tumor site. (**b**) Fh NPs in the context of drug delivery and cancer treatment (based on a schematic from Liu et al. [99]).

Biomedical research concerning Fh NPs is extensive but not yet comprehensively investigated. Iron-containing NPs are generally known for their enzyme-like activity, magnetothermal conversion capabilities, and enhanced Fenton reaction effects. In addition to their remarkable general catalytic properties [98], Fh and various Fh-based nanomaterials have been shown to exhibit strong enzyme-like activity in vivo. Fh catalyzes the decomposition of hydrogen peroxide into molecular oxygen in hypoxic tumor tissue, mitigating hypoxia and enhancing radiotherapy efficacy (Figure 8a) [8]. The selective treatment of tumors is based on the localized induction of apoptosis, which inhibits tumor growth by generating hydroxyl radicals at the tumor site (Figure 8b) [99].

The oxidative properties of Fh determine its efficacy as an antibacterial agent. Fh has demonstrated beneficial effects in the treatment of infected wounds [100]. Fh NPs loaded with antibiotics should be further assessed in relation to the management of inflammatory and wound-related conditions [101]. Fh should be further investigated for ferroptosis therapy [102], for catalytic treatment of rheumatoid arthritis [103], to develop nanozyme systems for inflammation therapy [104], and as a prospective antitumor agent [105]. Previous findings on Bio-Fh NPs in the context of drug delivery systems for medicine [106,107], binding to human serum transferrin [108], and influence on kidney and liver morphology [109,110] require further investigation.

Generally, in vitro and in vivo studies indicate that one of the mechanisms underlying the biological activity of Fh involves the induction of oxidative stress: specifically, an increase in the production of ROS [111,112,113]. ROS signaling regulates epigenetic modifications and the remodeling of gene expression patterns associated with cellular differentiation, proliferation, migration, and apoptosis. These cellular behaviors play a crucial role in tissue regeneration. The accelerated healing of superficial wounds in laboratory animals following Fh treatment [27,101] may be due to the influence of Fh on ROS signaling pathways.

Excessive ROS production is accompanied by destructive oxidative modifications of lipids, proteins, and DNA, leading to generalized dysfunction of membrane systems, disruption of signaling cascades and transport systems, and the development of cyto-genotoxic stress. This explains the observed toxicity of Fh as an inducer of oxidative stress. Cytotoxic effects have been demonstrated, with degenerative and necrotic changes observed in cell populations across various organs and tissues in laboratory animals [72,106,109,110]. One approach to mitigating the cytotoxicity of Fh involves the use of stable Fh–protein complexes. Mechanisms of Fh interaction with protein molecules via molecular docking require further research on the most promising proteins [55,82,107,108].

At the same time, the cytotoxicity of Fh determines its potential for use in cancer therapy [103,112]. The nonspecific nanotoxicity of Fh necessitates the development of targeted delivery systems to transport it to tumor tissues. In addition to nonspecific nanotoxicity, Fh has been shown to induce ferroptosis—an iron-dependent form of programmed cell death associated with the accumulation of lipid peroxides [102], a promising strategy for anticancer therapy. Yang et al. [114] employed photoresponsive Cu-Fh NPs to elucidate the photoregulatory mechanism of MHC-I in melanoma cells. They proposed blue-light-driven photoimmunotherapy with blue-light-activated photodynamic therapy for melanoma management and metastasis prevention. Pashkunova-Martic et al. [115] assessed Fh-salinomycin (Sal-Fh) composites in vitro as promising agents for antitumor activity.

To develop clinically effective and safe therapeutic agents based on Fh nanoparticles, it is essential to investigate the mechanisms of Fh interaction with both normal physiological cells and pathologically altered cells. Such a differentiated approach will enable the design of therapeutic Fh nanoformulations capable of exerting their biological activity specifically within cells exhibiting particular pathological changes.

Current biomedical research involving magnetic properties of NPs generally and Fh in particular focuses primarily on developing innovative agents for magnetic hyperthermia, targeted drug delivery systems for chemodynamic therapy, and MRI contrast agents [9,115]. Future research could lead to the development of targeted correction technologies addressing Fh-related protein misfolding, the targeted inhibition of amyloid fibril self-assembly, and the activation of amyloid disaggregation in vivo in the context of various conformational pathologies. Magnetic field effects should be further studied in view of recent progress observed on various NPs. It has been demonstrated that magnetic fields induce rearrangements of lipid rafts, i.e., structures formed through the lateral phase segregation of lipids with differing molecular morphologies, in biological membranes. This structural reorganization of lipid rafts alters the activity of raft-dependent endocytosis [116]. Magnetic fields have also been shown to induce phase transitions in standard bilayer (lamellar) lipid membranes, leading to the formation of non-bilayer structures (such as hexagonal phases) [117] and subsequently altering membrane transport activity [118]. Furthermore, weak magnetic fields influence protein folding and misfolding, as well as the self-assembly of protein subunits into organized cytoskeletal elements and pathogenic amyloid fibrils [119,120]. It has been demonstrated that a magnetic field inhibits Aβ protein aggregation in neurons and restores cognitive functions in vivo [121]. The regulatory effects of magnetic field fluctuations are mediated by cellular magnetosensory signaling cascades [122]. Fh NPs can also exert their biological activity through mechanosensory signaling systems. The activation of mechanosensory signaling depends on local membrane deformations resulting from interaction with the nanoparticles, namely local changes in curvature. A future direction is magnetic reprogramming: it has been shown that, in human stem cells, paramagnetic Fh undergoes biodegradation, and the resulting iron is subsequently used for the intracellular *de novo* synthesis of superparamagnetic particles [123].

While Fh NPs demonstrate significant promise for a wide range of potential future biomedical applications, a more comprehensive understanding of the mechanisms by which Fh NPs interact within the organism and studies on the precise determination of appropriate dosages are crucial. Studying the mechanisms underlying the biological activity of Fh nanoparticles under both physiological and pathological conditions will enable the development of biologically grounded engineering strategies for Fh-based agents tailored to specific clinical applications.

### 5.2. Fh in Nano-Agrotechnologies: Fh-Based Fertilizers, Adaptogens, and Pesticides

Various Fe-based NPs have been shown to stimulate plant species, including agricultural crops (e.g., [124,125]) due to the rapid transport into plant tissues and efficient delivery of Fe to the cellular level [126]. Associated with NPs is the advantage of bioavailability and minimizing the leaching of excess agrochemicals [127], as well as overcoming adverse conditions. Challenges due to more frequent temperature anomalies and droughts, the accumulation of agrochemicals, soil pollution, and plant diseases limit projected crop yield growth [128]. For example, plant diseases cause global yield losses that can reach 23% annually [129]. NPs can act as eustress agents [19,130], and at the end of the stress period, the adaptation is subsequently maintained [131]. Numerous laboratory and field studies demonstrate the positive effects of Fe nanoforms on various crop species [124,125,126], demonstrating higher uptake efficiency compared to traditional forms of Fe-containing fertilizers [12,132].

**Figure 9:**
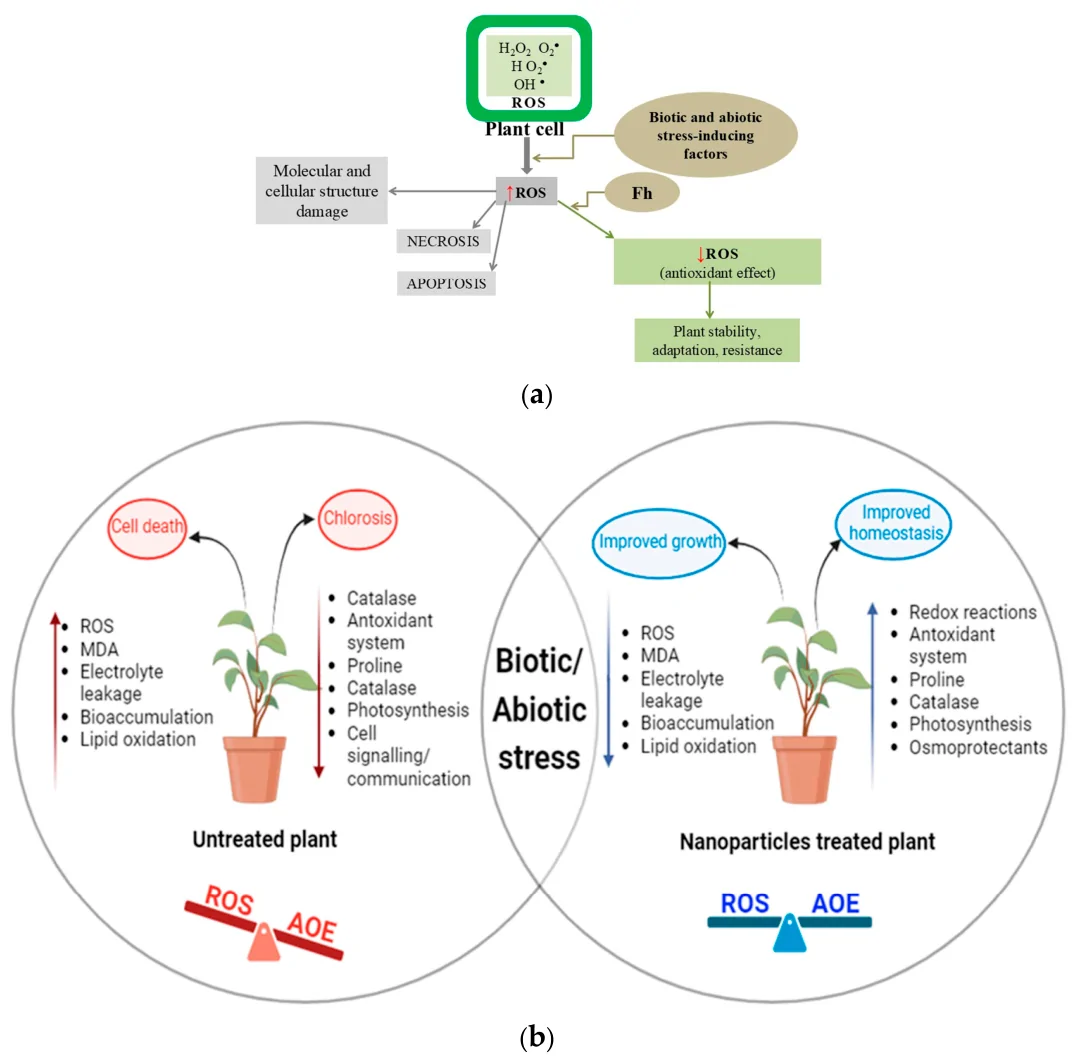
Schematic effects of ROS in plants. (**a**) High levels cause damage or cause necrosis/apoptosis, whereas moderate levels (modulated by Fh) are indicative of plant stability (prepared by the authors). (**b**) Improved growth of plants upon treatment with nanoparticles in oxidative stress leading to ROS–AOE (antioxidant enzyme) homeostasis (adopted from [135]). Depicted on the left is anbalanced situation (red arrows), on the right—balance with improved growth and homeostasis.

Fe-containing nanofertilizers are promising due to their high biological effect and low application rates [132], with the mechanisms attributed to ROS. Despite their potential for inducing cellular damage, the maintenance of their regulated concentrations is essential for normal growth and development [133]. ROS are functionally important signaling molecules that initiate root elongation and promote overall plant development [134], induce accelerated growth, increased biomass production and chlorophyll levels, optimized metabolic processes, augmented antioxidant capacity, and upregulated expression of stress-responsive genes [135]. Depending on their concentration in the plant, the role of ROS can be both positive and negative (Figure 9).

It has also been demonstrated that Fe nanoforms can exert their activity as nanozymes within plant cells [136]. The development of modeling methods for screening the biological activity of nanoparticles and nanomaterials is critical, as emphasized by Wei et al. [128], because the overwhelming diversity of different types and modifications of NPs precludes direct testing.

Future sustainable development of agriculture is linked to innovative nano-agrotechnologies employing nanoparticles as:

– Nanofertilizers featuring controlled, sustained release of micro- and macro-nutrients, high plant uptake efficiency, and low environmental risk (due to reduced effective concentrations); these are effective for specific crop types in certain soil types and help maintain soil fertility and the species diversity of soil microbiomes [126,132].

– Nano-adaptogens that enable the maintenance of high crop yields under adverse climatic conditions and in soils with high levels of phytotoxicant contamination [18,125,130,131,134].

– Nano-pesticides for protection against phytopathogens and pests, as well as for the activation of immune defense systems [128,129].

Foliar and root application of Fe nanoforms increases chlorophyll content and photosynthetic efficiency, stimulates root and shoot growth, and consequently enhances crop yield [16,127,129,134,137,138]. Furthermore, Fe nanoforms help maintain high yields under adverse abiotic conditions and improve resistance to various infections [125,129].

The potential for using Fh in cultivation technologies on soils heavily contaminated with phytotoxicants (heavy metals, arsenic, and organic compounds) stems from its high specific surface area (200–400 m^2^/g) and reactive surface groups [139,140,141,142,143,144,145]. The effectiveness of Fh application depends on irrigation regimes and soil geochemical characteristics [144,145,146]. The sorption capacity of Fh allows it to serve as an effective trap for chemical pesticides, preventing their leaching from the soil into groundwater and surface water [142].

The efficacy of Fh has been demonstrated for various monocot and dicot crops [147,148,149,150,151,152,153,154,155,156]. The use of Fh either in its pure form or as complex formulations (such as Fh–humic acid complexes) enables the effective correction of Fe deficiency in monocot and dicot plants [151,152,153]. While Fh itself does not exhibit intrinsic antifungal activity, it has been shown to enhance the efficacy of fungicides [157,158].

**Figure 10:**
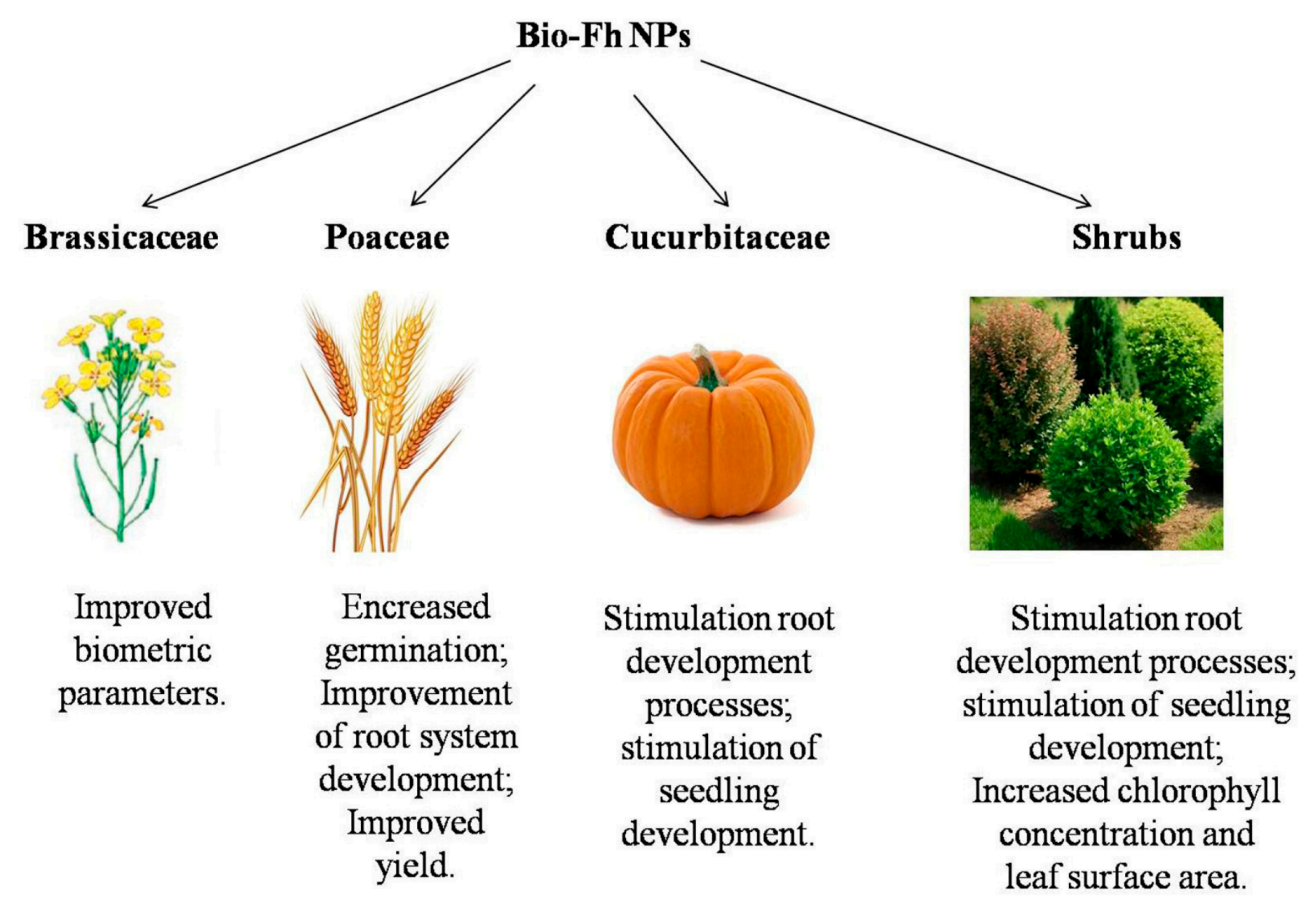
Demonstrated plant stimulation by Bio-Fh.

In case of infection of wheat (*T. aestivum* L.) grain with Alternaria, the use of Bio-Fh enhances the fungicidal effect of a preparation containing thiabendazole and tebuconazole [157]. Treatment of wheat seeds with a fungicide with the addition of Bio-Fh leads to a decrease in infestation [158]. The effect of Bio-Fh on three key stages of wheat callus culture development has also been studied: callus induction, callus proliferation, and organ regeneration. Stimulation of highly regenerative callus formation was demonstrated in a model system under study in [159]. Bio-Fh treatment increased the chlorophyll *b* content in blackcurrant leaves and the assimilation surface area of *Ribes nigrum* L. seedlings and led to an increase in the leaf surface area of plants [160]. The role of the interaction of NPs with phytohormones, as well as the role of ROS, was generally considered the underlying mechanism of action in the studies on Bio-Fh listed in Table S1 (Figure 10).

Overall, numerous studies demonstrate that Bio-Fh, in various doping modifications, significantly stimulates root development processes; it is highly effective as a priming agent [148] and as a stimulator of rhizogenesis both in vivo and in vitro (in callus cultures) [53,161,162,163,164,165]. Thus, the data presented in this section demonstrate the high biological activity of Fh NPs, manifested in the stimulation of biometric characteristics, increased biomass, modulation of biochemical parameters, and increased resistance of crop plants to stress-inducing factors. These effects offer significant potential for research into the use of Fh NPs in plant growth.

Future developments in Fe-nanotechnology focus on engineering complex agro-preparations that involve functionalizing nanoforms tailored to specific crop types and soils with distinct physicochemical properties.

## 6. Conclusions

Fh is an essential nanomaterial vital for iron storage and supporting essential biochemical processes in all organisms. Extensive research has been carried out over the years to elucidate the structural, morphological, and physical characteristics and the stability, size distribution, and magnetic properties of Fh of different origins. SAXS and SANS revealed that biogenic Fh NPs generated by *K. oxytoca* exhibit a cultivation-dependent architecture: short-term growth (Fe12) yields a two-level hierarchical structure (cylindrical subunits assembling into elongated aggregates), while prolonged growth (Fe34) produces a sponge-like surface fractal. Upon hydration, Fe12 particles undergo drastic restructuring, with the 1–2 nm minimal structural unit confirmed by both model-dependent fitting and ab initio reconstruction, as well as by HRTEM and magnetogranulometry.

Doping chemically synthesized Fh NPs with Co or Cu induces a core–shell architecture that fundamentally alters membrane interactions: undoped particles cause surface rigidification, while doped core–shell particles penetrate the bilayer; Cu-Fh NPs produce strong, persistent fluidization, and Co-Fh NPs show transient, cholesterol-dependent effects. SANS resolved the Fh NPs/LDH complex as a compact inorganic core surrounded by a protein corona with a rough fractal interface. This core–corona architecture rationalizes the entire set of binding and spectroscopic observations, as shown by Chilom et al. [83]. Although the organic corona is generally thought to increase biocompatibility, which is important for both biomedical and agricultural applications, future research is needed, as the imposed biochemical effects are complex: for instance, the hydrophilic outer layer of the water-soluble polysaccharide shell may facilitate the formation of colloidal solutions in aqueous media, enabling the transfer of catalytic activity. At the same time, the polar hydroxyl groups of these polysaccharides could compete with substrate hydroxyl groups, for example, those present in hydrogen peroxide, leading to the deactivation of Bio-Fh NPs.

The physicochemical properties of Fh determine several main areas of practical application: magnetic hyperthermia, targeted drug delivery systems for chemodynamic therapy, and MRI contrast agents and various agricultural technologies. One of the key mechanisms for Fh’s biological activity is associated with the induction of oxidative stress. Variability in the activity of Fh-dependent ROS production determines the potential for using Fh to stimulate tissue regeneration processes and to induce apoptosis and ferroptosis in oncotherapy. Studying Fh magnetosensory signaling will potentially enable the development of technologies for the targeted action of Fh magnetic fields on molecular events in cells in situ under normal and pathological conditions. The nanozyme activity of Fh highlights the potential for creating Fh-catalytic platforms for the treatment of inflammatory diseases. Numerous laboratory demonstrations demonstrate the feasibility of green Fh synthesis. The prospects for green synthesis lie in the development of algorithms for reprogramming the metabolism of producers to produce Fh nanoparticles with specific physicochemical characteristics adapted to specific tasks.

Overall, the major findings and directions can be summarized in the following ways:

1. Biomedical work on Fh covers therapy for melanoma and infected wounds, magnetically guided ampicillin delivery, nanozyme-based radical scavenging, and alleviation of oxidative stress and hypoxia in rheumatoid arthritis, as well as ferroptosis induction and the suppression of tumor proliferation. In agriculture, it restores iron deficiency, promotes seed germination and root development, and stimulates general plant development.

2. While effects are documented both for biomedical and agricultural applications, the comprehensive mechanistic framework and, especially, the prediction of the function should be subjects of future research. Future directions include exploring dose and exposure effects; unraveling the precise nanozyme activity mechanisms with reference to surface reactivity; systematic work on how the corona modifies membrane interaction and cellular uptake; biodegradability; and determining what fraction of the applied iron is actually active.

3. The structural and functional evidence indicates that Fh nanoparticles are promising across biological and agricultural applications, with the organic corona acting as a design element. Further progress depends less on identifying new applications than on quantifying existing observations.

## Figures and Tables

**Table 1:** Elemental contents of the Fe12 and Fe34 biogenic ferrihydrite samples determined by PIXE (percent dry weight). Data from [71].

Element	Fe12 wt.%	Fe34 wt.%
Al	0.327 ± 0.007	0.356 ± 0.009
Si	0.184 ± 0.008	0.233 ± 0.013
P	12.3 ± 0.1	15.1 ± 0.1
S	<0.02	0.16 ± 0.02
Cl	11.9 ± 0.1	1.19 ± 0.01
K	6.44 ± 0.02	4.11 ± 0.01
Ca	7.98 ± 0.03	6.67 ± 0.02
Cr	<0.01	0.027 ± 0.01
Mn	0.094 ± 0.003	<0.01
Fe	27.0 ± 0.1	32.7 ± 0.1
Zn	0.039 ± 0.010	<0.03
Rb	0.082 ± 0.016	0.247 ± 0.030
Hg	0.328 ± 0.036	0.684 ± 0.060
Pb	0.197 ± 0.035	0.494 ± 0.064

**Table 2:** Elemental contents of the Fe12 and Fe34 samples determined by PIGE (percent dry weight). Data from [71].

Element	Fe12 wt.%	Fe34 wt.%
Na	0.804 ± 0.040	0.234 ± 0.012
Mg	1.82 ± 0.60	1.10 ± 0.64
P	10.5 ± 0.5	11.1 ± 0.6
Cl	7.70 ± 1.00	0.89 ± 0.31
Fe	25.9 ± 1.8	30.9 ± 1.5

**Table 3:** Elemental contents of the Fe12 and Fe34 samples determined by RBS (percent dry weight). Data from [71].

Element	Fe12 wt.%	Fe34 wt.%
H	3.61	3.08
C	8.12	11.9
N	5.07	2.14
O	35.9	36.7
P	6.44	11.2
Cl	6.86	0.92
K	4.10	3.08
Ca	5.29	5.06
Fe	24.6	25.9

**Table 4:** SAXS structural parameters for biogenic sample Fe12 (two-level model).

Level	Model	Parameters(nm)	Physical Meaning
Level 1	Elliptical cylinder	a = 5.9 ± 0.6; H = 40.1 ± 3.8; ε = 2.9 ± 0.3	Elongated aggregates(a- semi-major axis; H- height; ε- eccentricity)
Level 2	Cylinder	R = 1.8 ± 0.01; H = 6.4 ± 0.02	Primary granular subunits(R-radius; H- height)

**Table 5:** SANS fitting parameters for chemically synthesized Fh-NPs (dry powders).

Parameter [nm]	Undoped Fh NPs	Co-Doped Fh NPs	Cu-Doped Fh NPs
Model	Triaxial ellipsoid	Core–shell ellipsoid	Core–shell ellipsoid
a/2	4.5 ± 0.2	58.7 ± 2.9	57.6 ± 4.8
b/2	54.9 ± 8.3	6.7 ± 0.5	7.1 ± 0.2
c/2	34.4 ± 4.1	–	–
Shell thickness	–	1.5 ± 0.2	1.8 ± 0.6
Morphology	Elongated clusters	Core–shell clusters	Core–shell clusters

**Table 6:** Structural parameters of the Fh NPs/LDH complex from SANS (triaxial ellipsoid core–shell model) obtained with FITTER.

Parameter	Value [nm]	Interpretation
Semi-axis (a/2)	10.4 ± 0.1	Core minor equatorial axis
Semi-axis (b/2)	77.7 ± 0.1	Core major equatorial axis
Semi-axis (c/2)	21.8 ± 0.1	Core polar axis
Shell thickness	322.8 ± 0.1	LDH protein corona
Overall dimension	<500	Fh NPs/LDH complex unit

## Data Availability

No new data were created during the course of preparing the present manuscript.

## Supplementary Materials

The following supporting information can be downloaded at: https://www.mdpi.com/article/10.3390/nano16201302/s1, Table S1: Testing the effects of Bio-Fh NPs on plants. References [54,155,156,157,158,159,160,161,162,163,164,165,166,167,168,169,170,171,172,173] are cited in the Supplementary Materials.
