# Supplementary material for: Examining Ferrihydrite Nanomaterials: From Structure and Biosynthesis to Biomedical and Agricultural Applications
Source: Nanomaterials (Basel). 2026 Oct 14;16(20):1302. doi: 10.3390/nano16201302 (PMC13707545; doi:10.3390/nano16201302)
Supplement: Supplementary file 1 [file nanomaterials-16-01302-s001.zip › nanomaterials-4537311-supplementary.pdf]

nanomaterials MDPI

Table S1. Testing the effects of Bio-Fh NPs on plants.

	Plants tested	Effect of NPs	References
1	*T. aestivum* seeds	Enhancement of the fungicidal action	[157,158]
2	*A. sativa* seeds	Encreased germination	[155]
3	*T. aestivum, A. sativa* seeds	Improved yield	[156]
4	*T. aestivum*	Improved induction of callusogenesis, callus proliferation	[159]
5	*R. nigrum* leaves	Improved leaf properties and chlorophyll content increase	[160]
6	*C. pepo* seedlings, cuttings of *P. fruticosa, T. occidentalis, R. nigrum, S. ledebouriana, P. coronarius, Cerasus fruticose* Pall, *Ribes atropurpureum* C.A. Mey, *Lonicera* L.	Stimulation root development processes, stimulation of seedling development	[54,161,162,163,164,165,166,167,168,169,170]
7	*Lepidium sativum* L.	Improved biometric parameters	[171]
8	*T. aestivum*	Rate of tissue aging decreased in callus	[172]
9	*R. nigrum* leaves	Increased chlorophyll concentration, leaf surface area	[173]

 https://doi.org/10.3390/nano16201302
